# STAT3 and mutp53 Engage a Positive Feedback Loop Involving HSP90 and the Mevalonate Pathway

**DOI:** 10.3389/fonc.2020.01102

**Published:** 2020-07-10

**Authors:** Maria Anele Romeo, Maria Saveria Gilardini Montani, Rossella Benedetti, Roberta Santarelli, Gabriella D'Orazi, Mara Cirone

**Affiliations:** ^1^Department of Experimental Medicine, “Sapienza” University of Rome, Rome, Italy; ^2^Laboratory Affiliated to Istituto Pasteur Italia-Fondazione Cenci Bolognetti, “Sapienza” University of Rome, Rome, Italy; ^3^Department of Research, IRCCS Regina Elena National Cancer Institute, Rome, Italy; ^4^Department of Medical, Oral and Biotechnological Sciences, University “G. d'Annunzio”, Chieti, Italy

**Keywords:** STAT3, mutp53, HSP90, mevalonate kinase, glioblastoma, pancreatic cancer

## Abstract

Oncosuppressor TP53 and oncogene STAT3 have been shown to engage an interplay in which they negatively influence each other. Conversely, mutant (mut) p53 may sustain STAT3 phosphorylation by displacing SH2 phosphatase while whether STAT3 could influence mutp53 has not been clarified yet. In this study we found that pharmacologic or genetic inhibition of STAT3 in both glioblastoma and pancreatic cancer cells, carrying mutp53 protein, reduced mutp53 expression level by down-regulating chaperone HSP90 as well as molecules belonging to the mevalonate pathway. On the other hand, HSP90 and the mevalonate pathway were involved in sustaining STAT3 phosphorylation mediated by mutp53. In conclusion, this study unveils for the first time that mutp53 can establish with STAT3, similarly to what observed with other oncogenic pathways, a criminal alliance with a crucial role in promoting cancerogenesis.

## Introduction

Signal Transducer and Activator of transcription (STAT) 3 is emerging as one of the most promising therapeutic targets in anti-cancer therapy, as its phosphorylation, especially in 705 tyrosine (Tyr705) residue, strongly contributes to cancer survival, progression and chemo-resistance (1). As such, STAT3 inhibition has been reported to trigger apoptosis in a variety of hematological and solid cancers displaying constitutive STAT3 activation (2), also inducing an immunogenic cell death type (3, 4). STAT3 may be activated by cytokines such as IL-6 and IL-10 that, released by tumor cells, besides acting in an autocrine fashion to sustain cancer cell survival, may activate this pathway in myeloid immune cells in the tumor environment, resulting in an impairment of their function (5). Therefore, targeting STAT3 may have a double beneficial effect, as it can act on both cancer and immune cell side. STAT3 has been reported to inhibit the expression of wtp53 at transcriptional level, as site-specific mutation in the STAT3 DNA-binding site in the p53 promoter partially abrogates such effect (6). The finding that STAT3 activation inhibits wtp53 is not surprising, as STAT3 is a pro-oncogenic molecule while wtp53 acts as a tumor suppressor molecule whose function is incompatible with cancer development. For this reason, p53 results mutated in over 50% of cancers and inhibited in the majority of the remaining ones. In line with these findings, we have shown that the inhibition of the constitutive STAT3 phosphorylation by Apigenin activated wtp53 to reduce Primary Effusion Lymphoma cell survival (7). More recently AG490 STAT3 inhibitor, by activating the p53-p21 axis, has been found to trigger KSHV replication in lymphoma cells harboring a latent viral infection (8). p53-p21 axis has been previously reported by ours and others' laboratories to be involved in KSHV lytic cycle activation (9, 10). Interestingly, other authors have reported that the wtp53 inhibited STAT3 activation, impairing its DNA binding activity (11), which suggests a reciprocal negative regulation between these two molecules. Less elucidated is the interplay between STAT3 and mutp53. This issue has been recently addressed by a study showing that, differently from wtp53, mutp53 sustained STAT3 phosphorylation by displacing the phosphatase SHP2 (12). However, whether STAT3 activation could influence mutp53 has not been explored yet. It is emerging that p53, as results of mutations that can occur at different sites, although more frequently in the DNA binding domain, not only loose its onco-suppressive capacity (LOS) but may also acquire oncogenic properties (GOF) (13). This is not due to the intrinsic characteristic of mutp53 but rather depends on its interaction with oncogenic pathways that mutp53 activates to increase its own stability. Among those, HSF1/HSP90 and the mevalonate pathways, both strongly involved in cancerogenesis (13). The HSF1/HSP90 pathway regulates the transcription of chaperoning molecules required for stabilization of oncoproteins and for helping cancer cells to cope with basal or induced cell stress (14) while the mevalonate pathway is needed for sterol and not-sterol isoprenoid production, required for post-translational modification of several proteins involved in cancerogenesis (15).

Interestingly, HSP90 (16) and molecules involved the mevalonate pathways (17) have been reported to be regulated by STAT3. Therefore, in the present study, we investigated whether STAT3 inhibition could reduce HSP90 and the mevalonate pathway molecules and through this mechanism reduce mutp53 expression level. As HSP90 and mevalonate pathway engage a cross-talk with mutp53 (13, 18), we then evaluated whether the downregulation of these molecules could contribute to the inhibition of STAT3 Tyr705 phosphorylation induced by mutp53 depletion. Finally, we evaluated whether STAT3 inhibition could impair cell survival and affect p53 and the mevalonate pathway in wtp53 carrying cancer cells.

## Materials and Methods

### Cell Cultures and Treatments

U373, T98G, and U87 (human glioblastoma cell lines with mutant and wild type p53) and Panc1 (human pancreatic cancer cell line with mutant p53) were grown in RPMI 1640 (Thermo Fisher Scientific), 10% Fetal Bovine Serum (FBS) (Corning), L- glutamine, streptomycin (100 μg/ml) (Corning), and penicillin (100 U/ml) (Corning) in 5% CO_2_ at 37°C. Cells were always detached using Trypsin-EDTA solution (Biological Industries, Cromwell, CT, USA).

U373, T98G, U87, and Panc1 cells were treated with AG490 (100 μM) (Millipore) for 48 h. U373 cells were treated with lovastatin (50 μM) (Sigma Aldrich) for 24 h. U373 cells were pre-treated with bortezomib (5 nM) (Santa Cruz Biotechnology) for 30 min and then treated with AG490 (100 μM) (Millipore) for 48 h. U373 and Panc1 cells were treated with geldanamycin (100 nM) (Sigma Aldrich) for 24 h.

### Trypan Blue Exclusion Assay

U373, Panc1, T98G, and U87 cells were plated in 6-well plates at a density of 2 × 10^5^ cells/well. The following day, when the cells were in the exponential growth phase, cells were treated with 100 μM AG490. After 48 h of culture, a trypan blue (Sigma Aldrich) exclusion assay was performed to test cell viability. Cells were counted by light microscopy using a Neubauer emocytometer. The experiments were performed in triplicate and repeated at least three times.

### STAT3 and p53 Silencing

1.5 × 10^6^ U373 cells were transfected with specific STAT3 siRNA transfection (Santa Cruz Biotechnology, sc-29493) and control siRNA-A (Santa Cruz Biotechnology, sc-37007) as a scrambled control for STAT3 knockdown, or with sip53 plasmid and empty vector as control for p53 knockdown, by electroporation using the Bio-Rad Pulse Controller at 180 V, according to the manufacturer's instructions, and cultured in 24 well plates. After 48 h cells were lysate and protein extracts were subjected to western blot analysis.

### Western Blot Analysis

1 × 10^6^ cells were washed with PBS and lysed in a RIPA buffer containing 150 mM NaCl, 1% NP-40 (Calbiochem), 50 mM Tris-HCl, pH 8, 0.5% deoxcycholic acid (SIGMA), 0.1% SDS, protease and phosphatase inhibitors. Twelve microgram of protein lysates were subjected to protein electrophoresis on 4–12% NuPage Bis-Tris gels (Sigma Aldrich). The gels were blotted on nitrocellulose membrane (Biorad) for 1 h in Tris-Glycine buffer. The membranes were blocked in PBS 0.1% Tween20 solution containing 3% of BSA, probed with specific antibodies and developed using ECL Blotting Substrate (Advansta).

### Antibodies

To evaluate the expression of proteins we used the following antibodies: mouse monoclonal anti-STAT3 (1:500) (BD Transduction Laboratories, 610,189), rabbit polyclonal anti-phospho-STAT3 (1:500) (p-Tyr705, clone D3A7, Cell Signaling Technology, 9145), mouse monoclonal anti-p53 (1:100) (clone DO-1, Santa Cruz Biotechnology Inc., sc-126), mouse monoclonal anti-HSP90 (1:100) (Santa Cruz Biotechnology Inc., sc-69703), mouse monoclonal anti-p21 (1:100) (clone F-8, Santa Cruz Biotechnology Inc., sc-271610), mouse monoclonal anti-SREBP1 (1:100) (clone A-4, Santa Cruz Biotechnology Inc., sc-365513), mouse monoclonal anti-MVK (1:100) (clone D-3, Santa Cruz Biotechnology Inc., sc-390669). Mouse monoclonal anti-β-actin (1:10,000) (Novus Biological, NB600-501) and goat polyclonal anti-lamin B (1:100) (Santa Cruz Biotechnology Inc, sc-374015) were used as loading control. The goat anti-mouse IgG-Horseradish Peroxidase (Santa Cruz Biotechnology Inc., sc- 2005), goat anti-rabbit IgG-HRP (Santa Cruz Biotechnology Inc., sc-2004) and rabbit anti-goat IgG-HRP (Santa Cruz Biotechnology Inc., sc-2768) were used as secondary antibodies. All the primary and secondary antibodies were diluted in PBS-0.1% Tween20 solution containing 3% of BSA (SERVA).

### Densitometric Analysis

The quantification of proteins bands was performed by densitometric analysis using the Image J software (1.47 version, NIH, Bethesda, MD, USA), which was downloaded from NIH website (http://imagej.nih.gov).

### Measurement of Intracellular Reactive Oxygen Species Production

To measure reactive oxygen species (ROS) production, 2,7-dichlorofluorescein diacetate (DCFDA; Sigma-Aldrich D6883) 10 μM was added to cell cultures for 15 min and live cells gated according to their forward scatter (FSC) and side scatter (SSC) properties were analyzed by FACScalibur flow cytometer (BD Transduction Laboratories), using CELLQuest Pro software (version 6.0, BD Biosciences). For each analysis 10,000 events were recorded.

### Statistical Analysis

Results are represented by the mean ± standard deviation (SD) of at least three independent experiments and a two-tailed Student's *t*-test was used to demonstrate statistical significance. Difference was considered as statistically significant when *p*-value was at least <0.05.

## Results

### Tyr705 STAT3 Inhibition by AG490 or STAT3 Silencing Reduce Cell Survival and mutp53 Expression Level in Glioblastoma and Pancreatic Cancer Cells Carrying R273 mutp53

**Figure 1:**
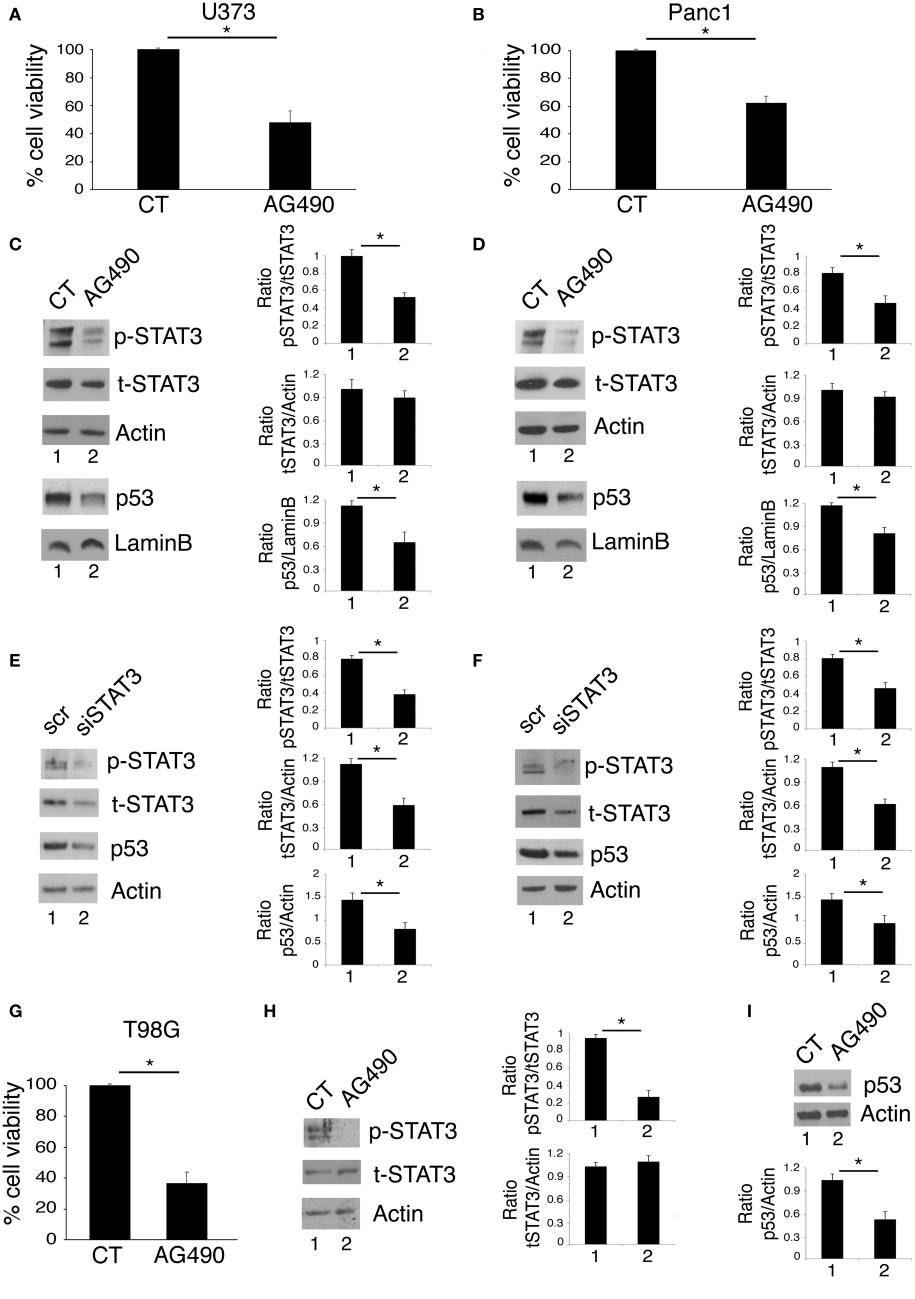
STAT3 inhibition reduces cell survival and mutp53 expression in U373 and Panc1 cell lines. U373, Panc1, and T98G cell lines were cultured in the absence or in the presence of 100 μM AG490 for 48 h, and cell survival and mutp53 expression together with p-STAT3 and t-STAT3 expression were studied by trypan blue exclusion assay **(A,B,G)** and by western blot **(C,D,H,I)**, respectively. The histograms **(A,B,G)** represent the mean plus S.D. of more than 3 experiments *P < 0.05; in western blot **(C,D)** Lamin B and **(H,I)** β Actin were used as loading control. In **(E,F)** mutp53 expression was evaluated by western blot in STAT3-silenced U373 **(E)** and Panc1 **(F)** for 48 h. β Actin was used as loading control. One representative experiment out of 3 is shown. The histograms represent the mean plus S.D. of the densitometric analysis of the ratio of specific band and control of 3 different experiments. *P < 0.05.

Targeting STAT3 has been shown to be an effective strategy to reduce the survival of several aggressive cancers that display constitutive Tyr705 STAT3 activation and p53 mutations, including glioblastoma (19) and pancreatic cancers (20, 21). Accordingly, in this study we found that the treatment with AG490 JAK2/STAT3 inhibitor reduced cell survival in glioblastoma (U373) and pancreatic (Panc1) cancer cell lines, harboring R273 hot spot mutation in DNA binding domain of p53 (Figures 1A,B). As mutp53 carrying cells strongly relay on its expression for their survival, we investigated whether AG490-mediated cytotoxicity could correlate with the reduction of mutp53 expression level. We found that the inhibition of STAT3 phosphorylation by AG490 down-regulated mutp53 in both cell lines (Figures 1C,D) and that also STAT3 silencing by specific siRNA reduced mutp53 expression level in U373 and Panc1 cells (Figures 1E,F). Finally, we used T98G glioblastoma cells, carrying M237I mutp53 and found that AG490 reduced cell survival and mutp53 expression also in these cells (Figures 1G–I), suggesting a more general effect link between STAT3 phosphorylation and p53 mutations.

### STAT3 Cross-Talks With the Mevalonate Pathway to Sustain mutp53 Expression Level

**Figure 2:**
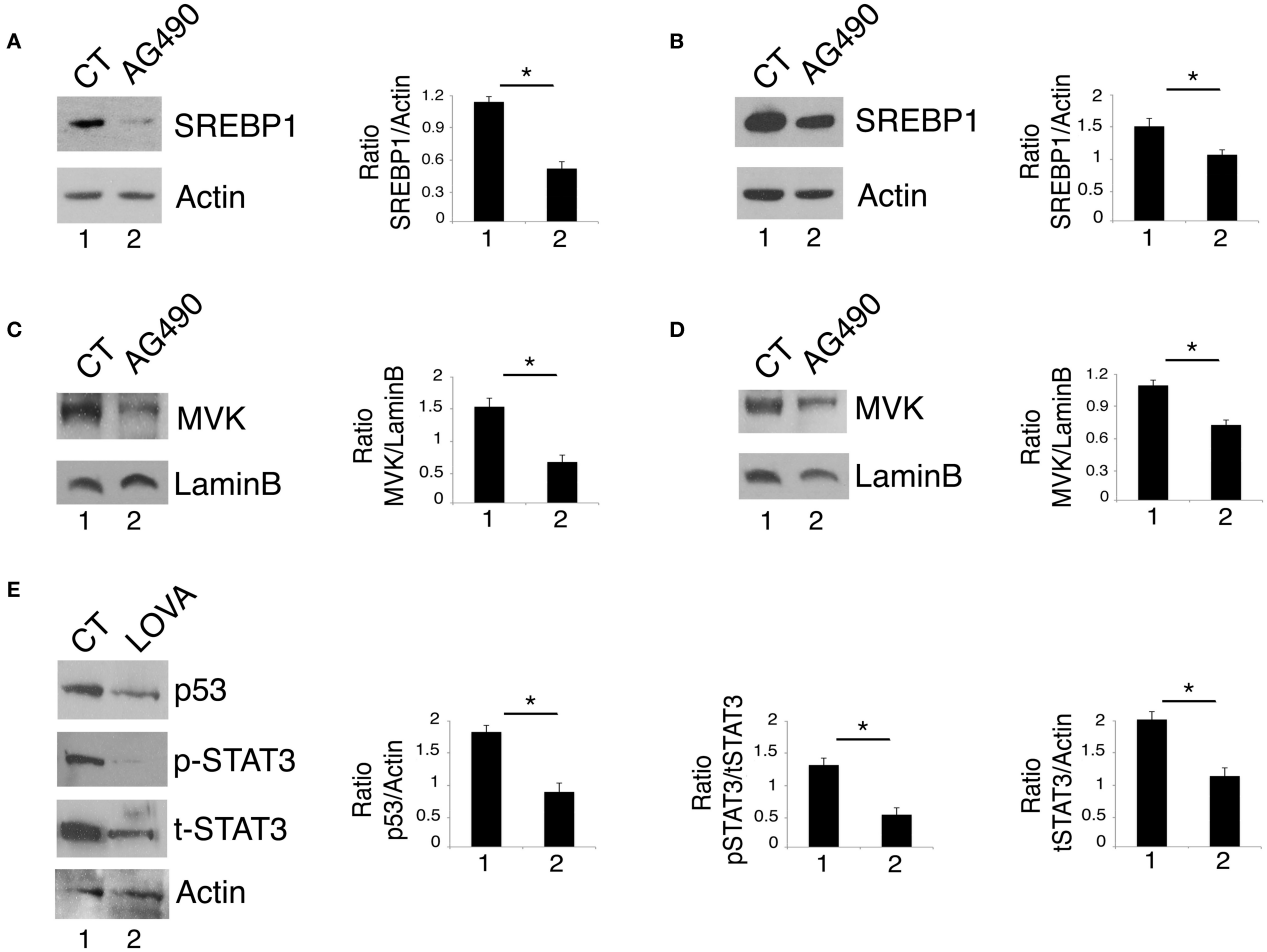
STAT3 cross-talks with the mevalonate pathway and sustains its interplay with mutp53. U373 **(A,C)** and Panc1 cells **(B,D)** cultured with 100 μM AG490 were analyzed by western blot for SREBP1 and MVK expression. In **(E)**, mutp53 and STAT3 expression of U373 cells cultured for 24 h with 50 μM lovastatin (LOVA) was analyzed by western blot. β Actin and Lamin B were used as loading control. One representative experiment out of 3 is shown. The histograms represent the mean plus S.D. of the densitometric analysis of the ratio of specific band and control of 3 different experiments. *P < 0.05.

By gene expression profiling approaches, it has been identified that STAT3 regulates the expression of the sterol regulatory element-binding proteins (SREBPs) and the transcription of the mevalonate cascade enzymes (22). Interestingly, the mevalonate pathway plays an important role in mutp53 stability, in a positive feedback loop (13, 23). Based on this knowledge, we next investigated whether STAT3 inhibition could affect the mevalonate pathway to down-regulate mutp53. At this aim, the expression level of SREBP1, one of the most important transcription factors controlling the transcription of the mevalonate enzymes and MVK, a key kinase of the mevalonate pathway, were investigated in U373 and Panc1 cells treated with AG490. The results shown in Figures 2A,B show that AG490 down-regulated SREBP1 in both cell lines and that also MVK expression level was reduced by such treatment (Figures 2C,D). The importance of the mevalonate pathway in down-regulating mutp53 expression in AG490-treated U373 cells was supported by the use of lovastatin, an inhibitor of the mevalonate pathway that efficiently reduced mutp53 expression level in these cells (Figure 2E). Interestingly, we found that lovastatin also inhibited STAT3 phosphorylation (Figure 2E) suggesting the occurrence of a cross-talk between STAT3 and the mevalonate cascade.

### STAT3/HSP90 Interplay Sustains mutp53 Expression Level and the Mevalonate Pathway

**Figure 3:**
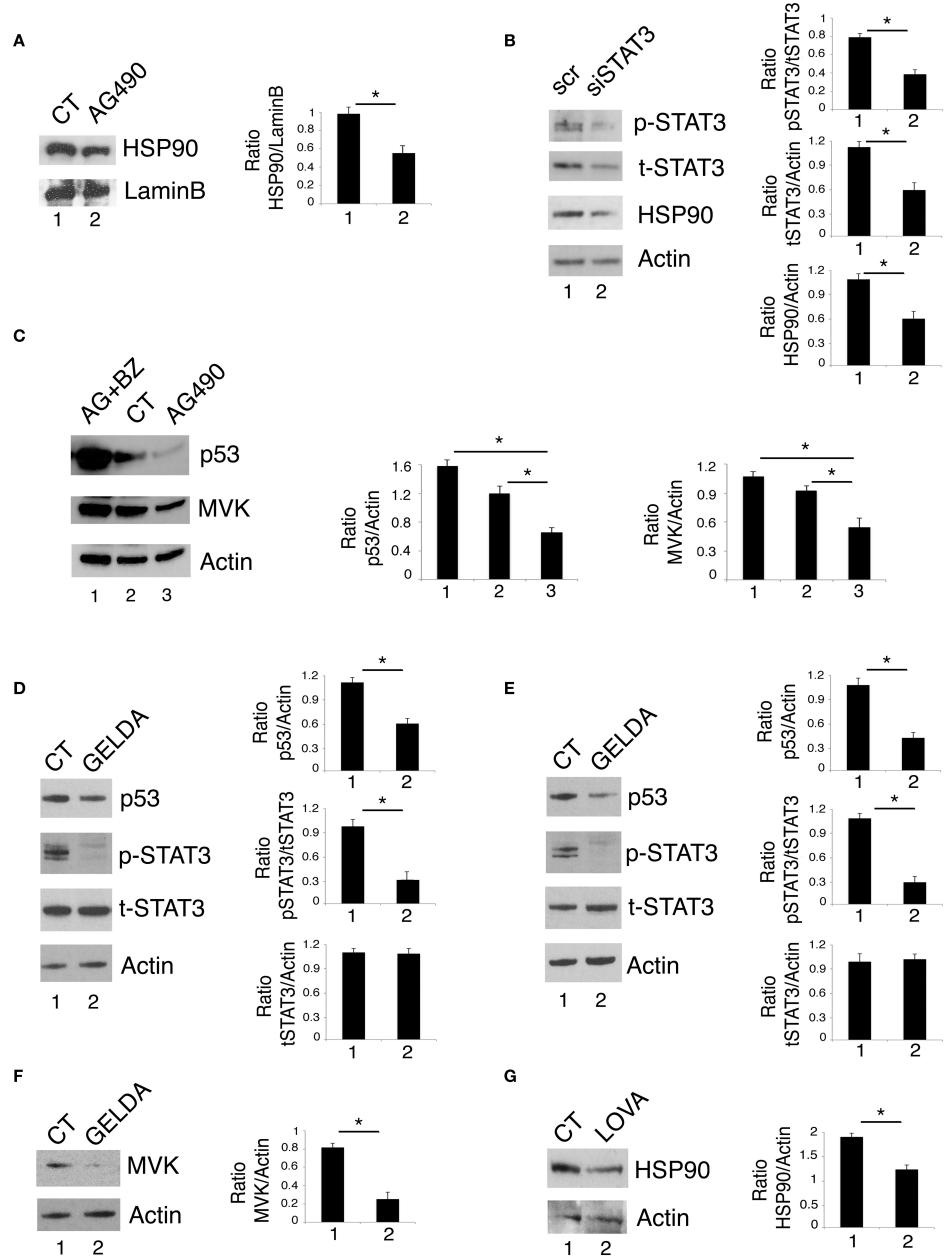
STAT3/HSP90 interplay sustains mutp53 expression level. U373 cell line, cultured with 100 μM AG490 **(A)** or STAT3 silenced **(B)** for 48 h, was analyzed by western blot for HSP90 protein expression. Lamin B and β Actin were used as loading control. In **(C)**, U373 cells pre-treated or not with 5 nM of bortezomib (BZ) and then treated with 100 μM AG490 for 48 h were analyzed by western blot for the expression of p53 and MVK. In **(D–F)** U373 **(D,F)** and Panc1 **(E)** cells were cultured with 100 nM geldanamycin (GELDA) and the expression of mutp53, STAT3, and MVK **(F)** was evaluated by western blot. In **(G)**, the expression of HSP90 in U373 cells cultured for 24 h with 50 μM lovastatin (LOVA) was analyzed by western blot. β Actin was used as loading control. One representative experiment out of 3 is shown. The histograms represent the mean plus S.D. of the densitometric analysis of the ratio of specific band and control of 3 different experiments. *P < 0.05.

Previous studies by our and other's laboratories have shown that inhibition of STAT3 down-regulated HSP90 expression in cancer cells (2, 16). As mutp53 is highly dependent on HSP90 for its stability (23–25), we then investigated whether the reduction of mutp53 expression level induced by STAT3 inhibition could correlate with the down-regulation of HSP90. As shown in Figures 3A,B, HSP90 expression level was reduced by AG490 as well as by STAT3 silencing by specific siRNA. These results suggest that the reduction of mutp53 expression level mediated by STAT3 inhibition was involved the down-regulation of HSP90. To evaluate whether the reduction of mutp53 was due to its proteasomal degradation, we used the proteasome inhibitor bortezomib and found that mutp53 accumulated when this drug was used in combination with AG490 (Figure 3C). Interestingly, bortezomib treatment, together with mutp53 also increased MVK expression level (Figure 3C), further highlighting the correlation between the two molecules previously observed (26). The role of HSP90 in the stabilization of mutp53 in this setting was then confirmed by use of geldanamycin an HSP90 inhibitor that strongly reduced mut53 expression level in both U373 and Panc1 cell lines (Figures 3D,E). Interestingly, geldanamycin reduced also STAT3 phosphorylation (Figures 3D,E), suggesting that HSP90 may in turn sustain STAT3 phosphorylation, in a positive feedback loop crucial for mutp53 stability. Furthermore, geldanamycin reduced MVK expression (Figure 3F) and, on the other hand, lovastatin down-regulated HSP90 (Figure 3G) highlighting another important loop sustained by STAT3 activation.

### p53 Silencing Inhibits STAT3 Activation in Cancer Cells Carrying mutp53

**Figure 4:**
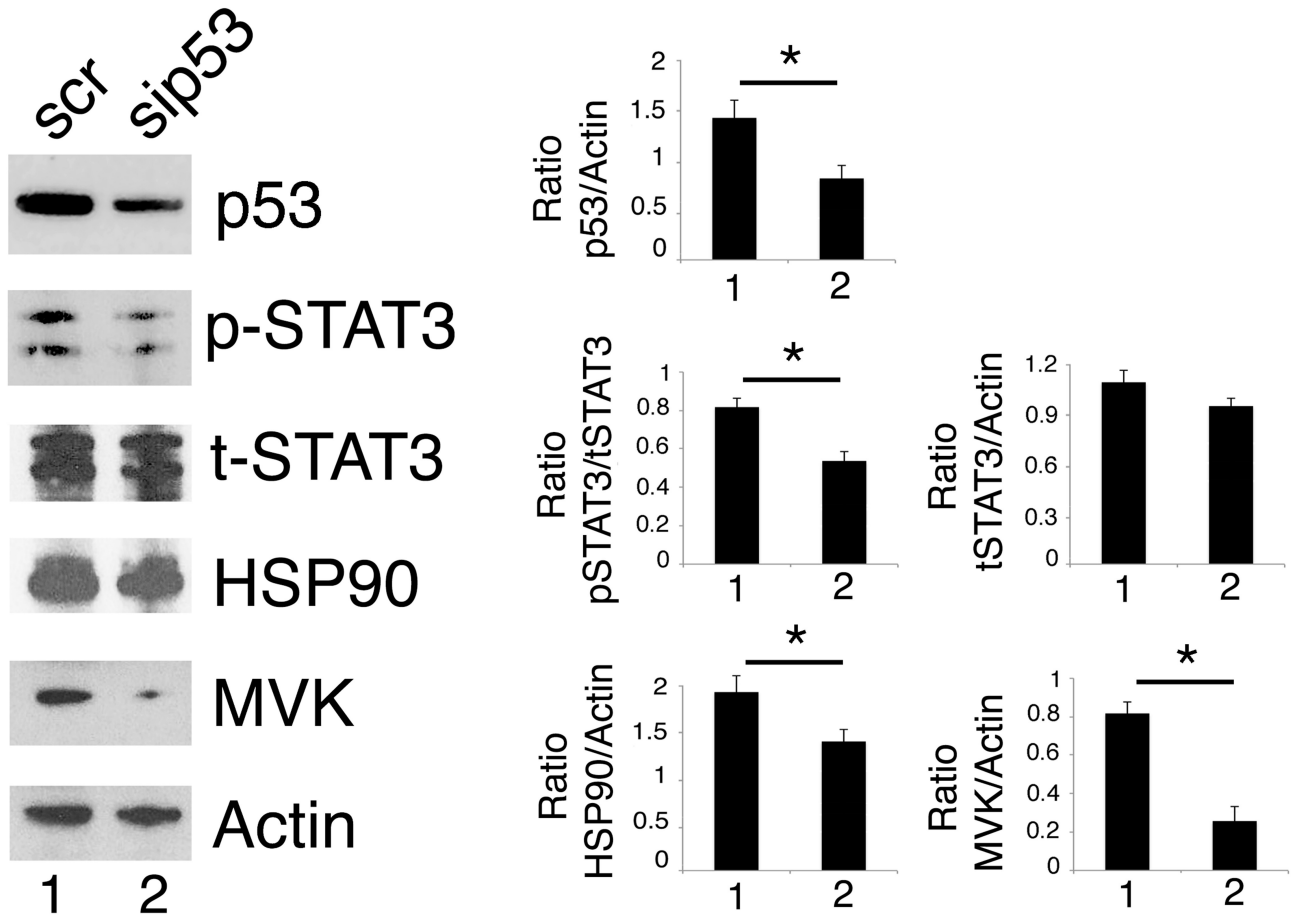
p53 silencing inhibits STAT3 and reduces HSP90 and MVK expression level. U373 cells silenced for p53 for 48 h were analyzed by western blot to evaluate mutp53, STAT3, HSP90, and MVK protein expression. β Actin was used as loading control. One representative experiment out of 3 is shown. The histograms represent the mean plus S.D. of the densitometric analysis of the ratio of specific band and control of 3 different experiments. *P < 0.05.

As mutp53 has been reported to sustain Tyr705 STAT3 phosphorylation in colon cancer cells (12), here we evaluated whether the silencing of R273 mutp53 could reduce the constitutive Tyr705 STAT3 phosphorylation also in our experimental conditions. As shown in Figure 4, mutp53 knocking down reduced STAT3 activation in U373 cells, confirming results previously obtained in other cell types.

We then found that the silencing of mutp53 reduced HSP90 and MVK expression (Figure 4), according to the positive feedback loops reported to occur between mutp53 and HSP90 or mutp53 and the mevalonate pathway (23, 27). Therefore, HSP90 and MVK down-regulation induced by mutp53 could contribute to the inhibition of STAT3 phosphorylation, in addition to the previously reported displacing of SH2 phosphatase (12). The above reported results indicating that geldanamycin (Figures 3D,E) or lovastatin (Figure 2E) were able to reduce Tyr705STAT3 phosphorylation, supporting the possibility that the reduction of HSP90 and MVK could be involved in STAT3 inhibition mediated by mutp53 depletion.

### STAT3 Inhibition Reduces Cell Survival by Activating p53 and Down-Regulating MVK in Glioblastoma Cancer Cells Carrying wtp53

**Figure 5:**
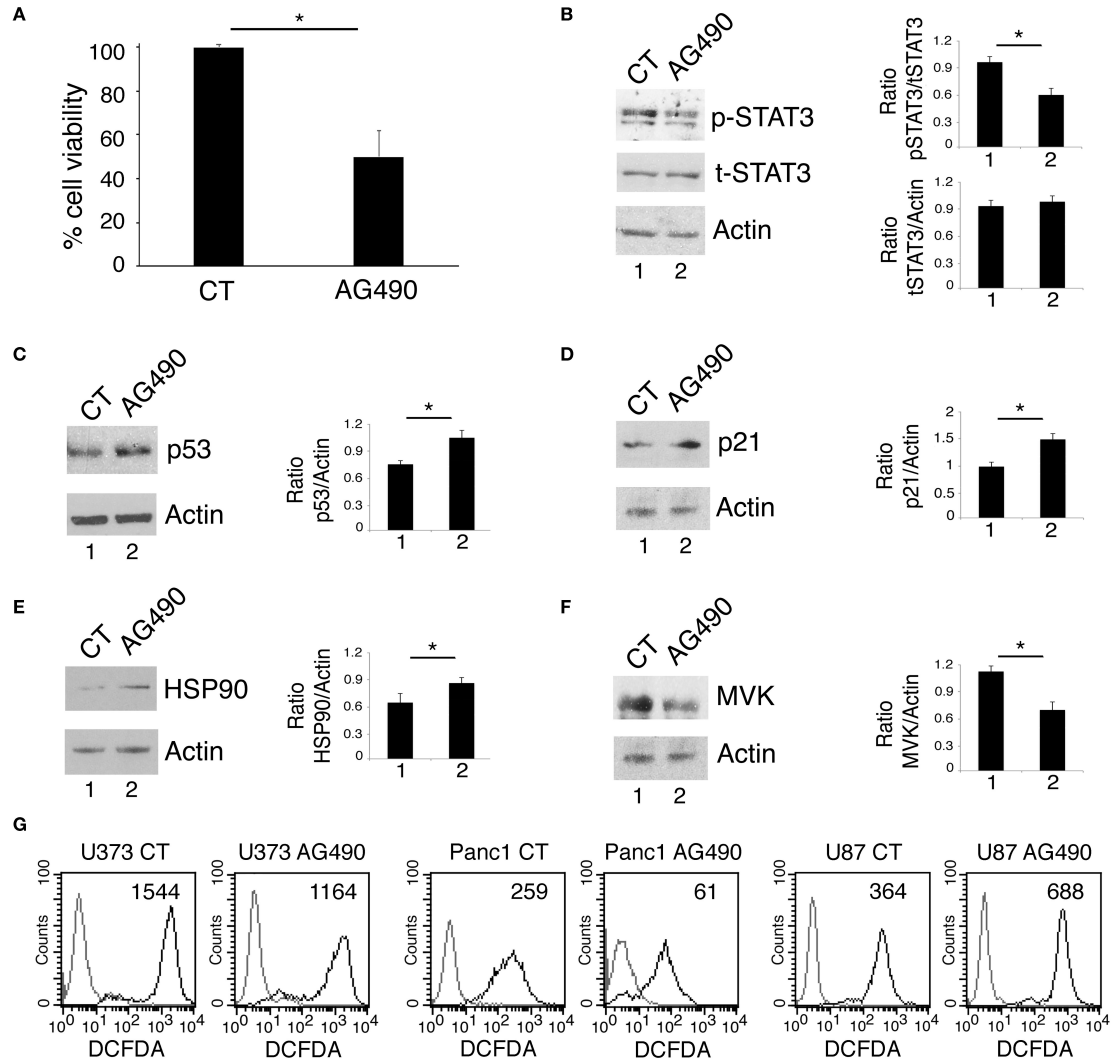
STAT3 inhibition in wtp53 U87 cells reduces cell survival and increase p53 and p21 expression and inhibits the mevalonate pathway. U87 cells were cultured in the absence or in the presence of 100 μM AG490 for 48 h, and cell survival and STAT3, p53, p21, HSP90, and MVK expression were analyzed, respectively, by trypan blue exclusion assay **(A)** and by western blot **(B–F)**. The histograms **(A)** represent the mean plus S.D. of more than 3 experiments *P < 0.05; in western blot **(B–F)** β Actin was used as loading control. One representative experiment out of 3 is shown. The histograms represent the mean plus S.D. of the densitometric analysis of the ratio of specific band and control of 3 different experiments. In **(G)**, FACS analysis of ROS production, by U373, Panc1, and U87 treated or not with 100 μM AG490, measured by DCFDA staining. The mean of fluorescence intensity is indicated. Solid gray peaks represent the controls. One representative experiment out of 3 is shown.

We next investigated whether AG490 STAT3 inhibitor could exert cytotoxic effects against U87 glioblastoma cells harboring wtp53. We found that such treatment reduced cell survival also in these cells (Figure 5A) and inhibited STAT3 phosphorylation (Figure 5B). However, differently from mutp53, AG490 up-regulated wtp53 (Figure 5C) and its target p21 (Figure 5D) in this cell line. Moreover, we found that AG490 treatment slightly up-regulated HSP90 in U87 cells (Figure 5E), effect that could contribute to p53 stabilization. We then asked HSP90 up-regulation in U87 cells could correlate with an increase of ROS in U87 cells undergoing AG490 treatment. As shown in Figure 5G, we found that intracellular ROS increased in U87 wtp53 carrying cells while decreased in mutp53 carrying cells undergoing AG490 treatment. Next, as oppositely from mutp53, wtp53 has been reported to inhibit the mevalonate pathway, we then evaluated whether the activation of wtp53 by AG490 could correlate with a reduced expression of MVK in U87 cells. As shown in Figure 5F, we found that MVK expression was reduced in U87 cells treated with AG490 suggesting that the reduction of the mevalonate pathway could play a role in the impairment of cell survival induced by AG490 that increased wtp53 expression level.

## Discussion

**Figure 6:**
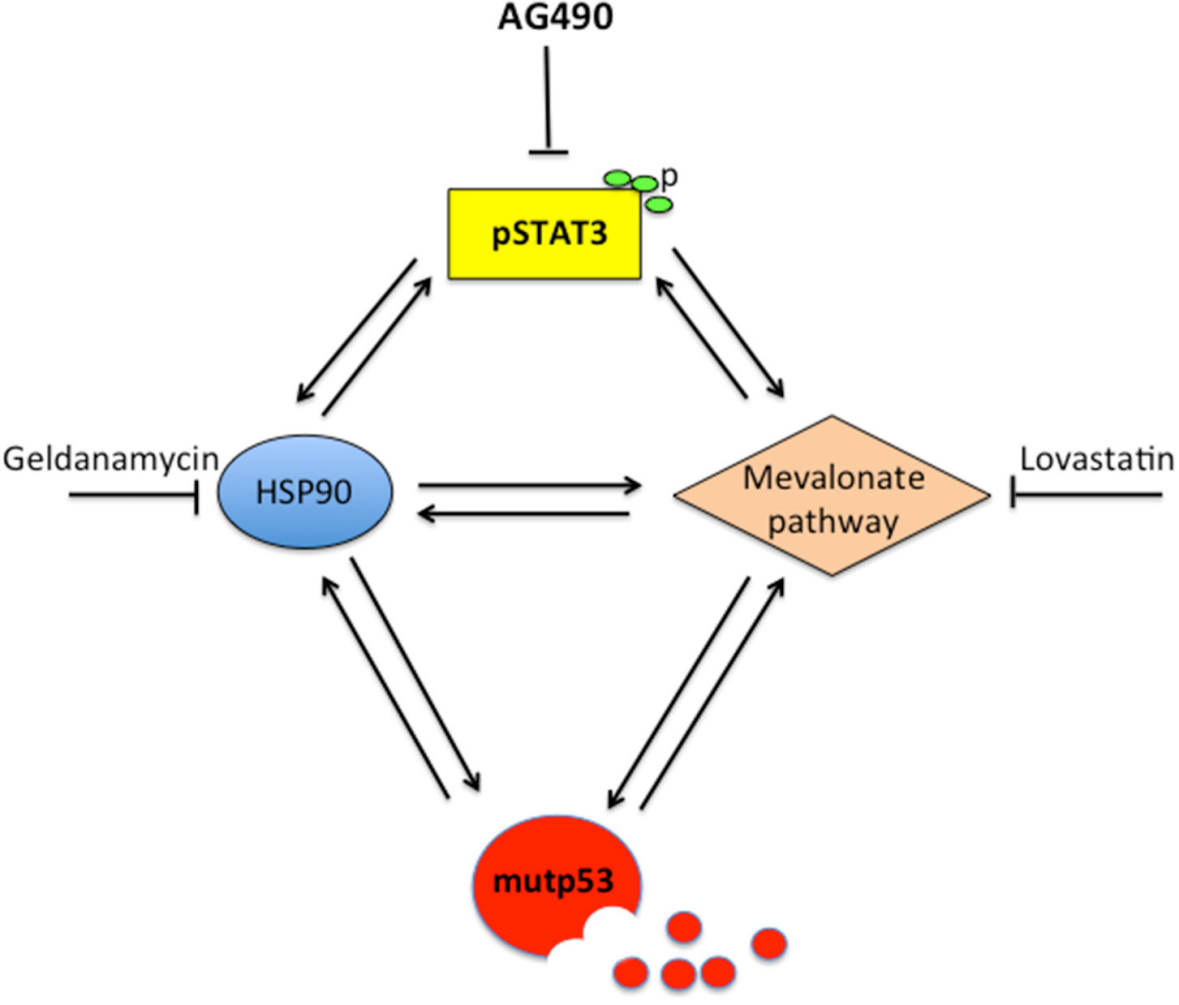
Scheme showing the capacity of STAT3 to regulate HSP90, the mevalonate pathway, mutp53 and the cross-talk between these molecules.

This study unveils for the first time that STAT3 sustains mutp53 expression level due to its interplay with HSP90 and the mevalonate pathway that increases its stability and prevents its degradation via proteasome. Previous studies have highlighted that HSP90 and the mevalonate cascade could increase mutp53 stability (13, 18, 23, 27, 28). Moreover, STAT3 has been reported to positively regulate HSP90 (2, 16) and SREBPs as well as several enzymes of the mevalonate pathway, including the mevalonate kinase MVK (22, 29, 30). Interestingly, HSP90 may in turn stabilize STAT3 (31) and maintain it phosphorylated by up-regulating the expression of JAKs, the most important kinases involved in Tyr705 STAT3 phosphorylation (32). The mevalonate pathway may also contribute to STAT3 Tyr705 phosphorylation, as indicated by the use of statins that reduced STAT3 activation (17). In the present study, we found that the expression of HSP90 and MVK was down-regulated by p53-silencing, suggesting that STAT3 inhibition by mutp53 could involve the down-regulation of these molecules. This hypothesis was supported by the finding that geldanamycin or lovastatin reduced STAT3 phosphorylation in both U373 and Panc1 cells. This could be a new mechanism involved in the regulation of STAT3 by mutp53, in addition to the displacement of STAT3 phosphatase SHP2, recently reported (12). More importantly, this study suggests that STAT3 and mutp53 establish another criminal alliance that promotes cancerogenesis. The interplay with pro-oncogenic pathways is fundamental for mutp53 stability and GOF. Besides HSF1/HSP90 and mevalonate, indeed mutp53 engages a positive feed-back loop with NRF2, the most important transcription factor regulating the antioxidant response, and with HIF, essential for the adaption of cancer cells to hypoxia conditions (13, 23). The cross-talk with STAT3, highlighted in this study, suggests that STAT3 could another pathway crucial for mutp53 GOF. STAT3 is indeed able to up-regulate a variety of molecules involved in cell proliferation and evasion from apoptosis, including c-myc, survivin, and cyclin D (33, 34). STAT3 has been previously reported to positively influence the mevalonate (22, 29, 30) and HSF1/HSP pathways (16). Therefore, based on this and previous studies, STAT3 can be consider at the center of a hub crucial for the control of tumorigenesis (Figure 6). Indeed, STAT3 activation sustains the interplay between HSP90 and the mevalonate cascade, according to previous studies showing that HSF1 sustained the mevalonate pathway (35), that HSP90 sustained SREBP activation (36) and that Simvastatin inhibited HSP90 (37).

Of note, STAT3 can be activated by several cytokines including VEGF, whose production is promoted by STAT3 activation (38) and also by mutp53 due to its interaction with NFkB (13). Therefore, the interplay between STAT3 and mutp53 could play a pivotal role to induce a pro-cancerogenic microenvironment. Interestingly, several pro-inflammatory cytokines, acting in an autocrine fashion on cancer cells, besides STAT3, may activate other pro-oncogenic pathways such as mTOR, crucial for cancer survival (39). The relation between mTOR and mutp53 has also been previously investigated by studies showing that mutp53 could activate mTOR to inhibit autophagy and prevent its own degradation (40). STAT3 could cooperate with mTOR in preventing mutp53 degradation by autophagy. In conclusion, differently from other drugs that preferentially kill mutp53 carrying cancer cells (41), we found that the cytotoxic effect of STAT3 inhibition goes behind its-capacity to down-regulate mutp53, as AG490 efficiently impaired cell survival also of wtp53 carrying glioblastoma cells, in correlation with p53 activation and the mevalonate pathway inhibition. Therefore, this study strongly encourages the targeting of STAT3 in anti-cancer therapy considering that, in addition to the inhibition of pro-survival molecules so far reported, our study suggests that STAT3 stabilizes mutp53 in cancer cells carrying mutp53 and activates wtp53 in cancer cells harboring wtp53.

## Data Availability Statement

The raw data supporting the conclusions of this article will be made available by the authors, without undue reservation.

## Author Contributions

MR, MG, GD'O, and MC: design, acquisition, analysis, and interpretation of data. MC: writing manuscript. MG and MC: editing manuscript. MR, MG, RB, and RS: methodology. All authors: revised the drafts and approved the manuscript submission.

## Conflict of Interest

The authors declare that the research was conducted in the absence of any commercial or financial relationships that could be construed as a potential conflict of interest.
